# Chemical Characteristics of *Platycodon grandiflorum* and its Mechanism in Lung Cancer Treatment

**DOI:** 10.3389/fphar.2020.609825

**Published:** 2021-02-12

**Authors:** Yaling Deng, Xianwen Ye, Yufan Chen, Hongmin Ren, Lanting Xia, Ying Liu, Minmin Liu, Haiping Liu, Huangang Zhang, Kairui Wang, Jinlian Zhang, Zhongwei Zhang

**Affiliations:** ^1^ School of Pharmacy, Jiangxi University of Traditional Chinese Medicine, Nanchang, China; ^2^ Patient Service Center, Ganzhou People's Hospital, Ganzhou, China; ^3^ School of Pharmacy, Guilin Medical University, Guilin, China; ^4^ School of Pharmacy, Youjiang Medical University for Nationalities, Guangxi, China

**Keywords:** lung cancer, molecular mechanism, network pharmacology, Platycodon grandiflorum, ultra performance liquid chromatography-quadrupole time-of-flight tandem mass spectrometry

## Abstract

**Objective:** The technology, network pharmacology and molecular docking technology of the ultra performance liquid chromatography-quadrupole time-of-flight tandem mass spectrometry (UPLC-Q-TOF-MS/MS) were used to explore the potential molecular mechanism of *Platycodon grandiflorum* (PG) in the treatment of lung cancer (LC).

**Methods:** UPLC-Q-TOF-MS/MS technology was used to analyze the ingredients of PG and the potential LC targets were obtained from the Traditional Chinese Medicine Systems Pharmacology database, and the Analysis Platform (TCMSP), GeneCards and other databases. The interaction network of the drug-disease targets was constructed with the additional use of STRING 11.0. The pathway enrichment analysis was carried out using Gene ontology (GO) and Kyoto Encyclopedia of Genes and Genomes (KEGG) in Metascape, and then the “Drug-Ingredients-Targets-Pathways-Disease” (D-I-T-P-D) network was constructed using Cytoscape v3.7.1. Finally, the Discovery Studio 2016 (DS) software was used to evaluate the molecular docking.

**Results:** Forty-seven compounds in PG, including triterpenoid saponins, steroidal saponins and flavonoids, were identified and nine main bioactive components including platycodin D were screened. According to the method of data mining, 545 potential drug targets and 2,664 disease-related targets were collected. The results of topological analysis revealed 20 core targets including caspase 3 (CASP3) and prostaglandin-endoperoxide synthase 2 (PTGS2) suggesting that the potential signaling pathway potentially involved in the treatment of LC included MAPK signaling pathway and P13K-AKT signaling pathway. The results of molecular docking proved that the bound of the ingredients with potential key targets was excellent.

**Conclusion:** The results in this study provided a novel insight in the exploration of the mechanism of action of PG against LC.

## Introduction

Lung cancer (LC) is a malignant tumor with the highest morbidity and mortality worldwide, mostly in male than in female, indeed known as “the king of cancer” (Shao and Zhang, 2020). At present, surgery, chemotherapy and radiotherapy are the main treatments to combat LC, but their side effects are numerous and unavoidable and the clinical prognosis is not ideal. The development of traditional Chinese medicine (TCM) included the discovery and used of anti-cancer drugs; thus, it has attracted more and more attention. TCM adopts syndrome specific treatments, TCM combined with chemotherapy and other methods, with less toxic side effects. For this reason, TCM prescriptions achieved good results in clinical practice (Bai et al., 2017; Wang and Li, 2019). Therefore, the research and development of new TCM to combat LC would be of great significance.

*Platycodon grandiflorum* (PG) is a plant belonging to the family of campanulaceae, and the dried root is used in TCM to regulate the lung meridian. PG exerts the effect of smoothing lung, dispelling the phlegm, and expelling the pus, and represents the main treatment to cure sore throat, vomiting due to a purulent carbuncle infection in the lung, hypochondriac pain in the chest and other syndromes (Chinese Pharmacopoeia Commission, 2020). The properties of PG were first published in Shennong Materia Medica Classic. PG is mainly growing in the Northeast China, Central China and Guangdong, and the components of PG are different in different areas. Shandong is one of the authentic areas cultivating PG. The roots of PG from Shandong are longer, less bifurcated and with a high content of active components (Zhu et al., 2013). According to the TCM, PG mainly acts on the lung and its related parts, with an antitussive and expectorant effect, and good therapeutic effect on LC (Yim et al., 2016; Li et al., 2019; Deng et al., 2020). However, its mechanism of action in the treatment of LC is not clear.

**FIGURE 1:**
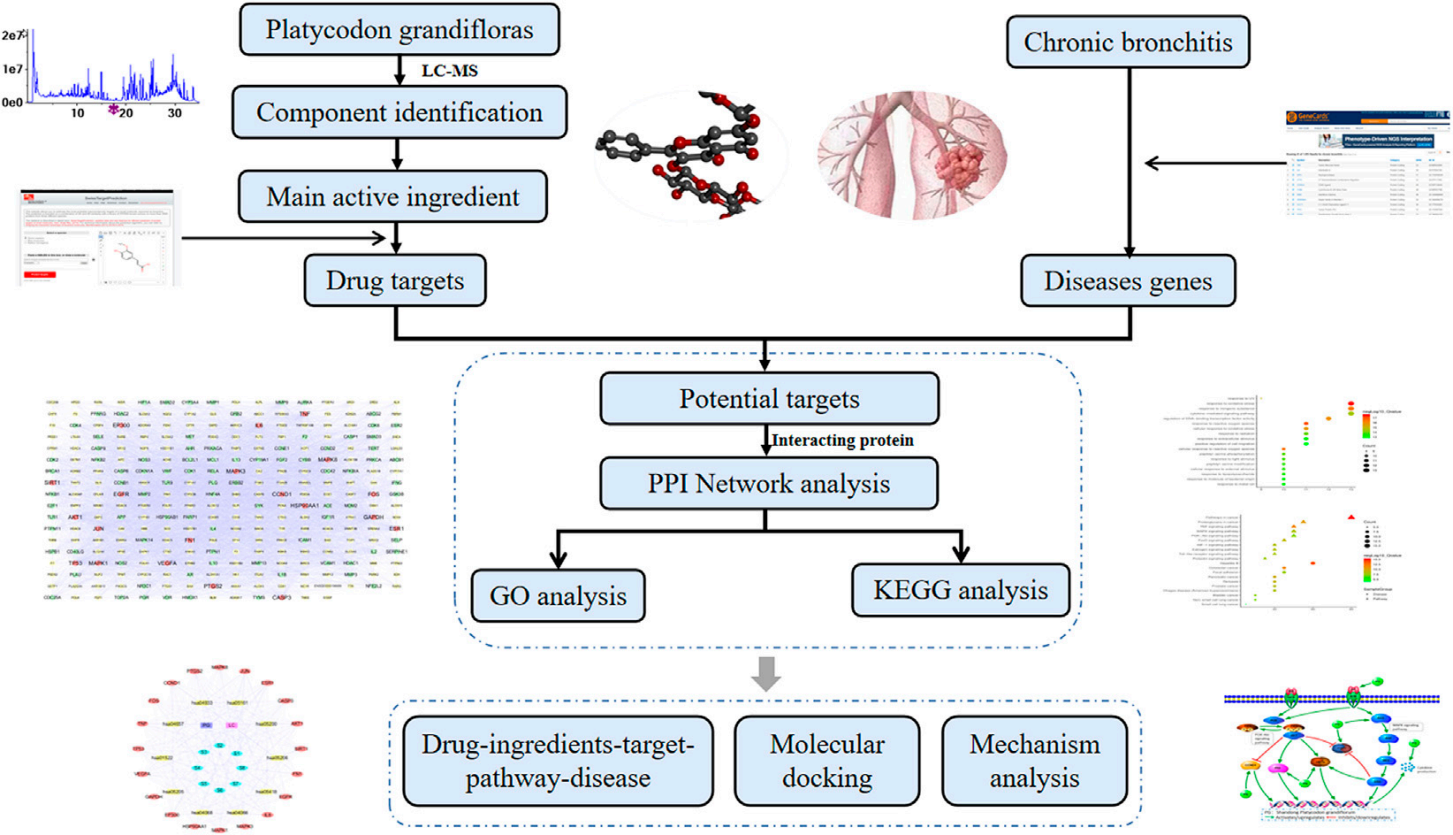
A comprehensive strategy diagram the chemical ingredients analysis, targets prediction, and network calculation for investigation the mechanism of action of *Platycodon grandiflorum* on lung cancer.

UPLC-Q-TOF-MS/MS is a high-throughput analytical technology rapidly developed in the past decade, which is widely used in medicine, drug research and other fields (Jin et al., 2018; Ren et al., 2020). Network pharmacology is a theory based on systems biology, which emphasizes the multi-pathway regulation of signaling pathways, thus in agreement with the multi-component-multi-target characteristics of TCM (Yang et al., 2019; Zhang et al., 2019; Ye et al., 2020). Therefore, in this study, the components of the PG roots cultivated in the Shandong Province were analyzed, and then the “Drug-Ingredients-Targets-Pathways-Disease” (D-I-T-P-D) network was constructed according to the relevant principles and methods of network pharmacology to explore the potential molecular mechanism used by PG to treat LC. Our aim was to identify a new drug through the development of the potential of PG to provide a theoretical basis for its clinical application. The flow chart of the approach used in this study is shown in Figure 1.

## Materials and Methods

### Chemicals and Materials

Methanol, acetonitrile, and formic acid used for high performance liquid chromatography (HPLC) were purchased from ACS (Washington D.C., MD, United States). Methanol for herb extraction was purchased from Xilong Scientific Co., Ltd (Guangdong, China). Ultrapure water was obtained from a Milli-QB system (Bedford, MA, United States). PG root pieces (simply defined as PG pieces according to the Chinese Pharmacopoeia 2015 edition that assumes that PG is the root) were purchased from Jiangxi Jiangzhong Herbal Pieces Co., Ltd (Jiangxi, China; batch number: 181024). The original PG medicinal material was purchased from Yiyuan, Shandong province, and was identified as the dried root of *Platycodon grandiflorum* (Jacq.) A. DC. Campanulaceae from Professor Fu Xiaomei. PG decoction pieces were processed by Jiangxi Jiangzhong TCM Decoction Co., Ltd. according to the processing method of the Chinese Pharmacopoeia 2015 edition. Next, dried PG pieces were crushed into a 40 mesh powder and stored in the laboratory of the Jiangxi University of TCM.

A total of 15 pure compounds were used as reference standards (purity≥98%). Among them, deapio-platycodin D, platycodin D, polygonatoside C1, rubinin, luteolin, kaempferol, apigenin, caffeic acid, ferulic acid, adenosine, and lobetyolin were purchased from Chengdu Chroma-Biotechnology Co., Ltd (Sichuan, China). 3-O-β-D-glucopyranosyl platycodigenin, rutin, chlorogenic acid, and linoleic acid were purchased from Sichuan Vicky Biotechnology Co., Ltd (Sichuan, China).

### Ultra-Performance Liquid Chromatography-Quadrupole-Time-Of-Flight Tandem Mass (UPLC-Q-TOF-MS/MS) Analysis

#### Preparation of Standard and Sample Solutions

Ten milligrams of each reference compound (deapio-platycodin D, platycodin D, polygonatoside C1, adenosine, ferulic acid, apigenin, luteolin, chlorogenic acid, caffeic acid, kaempferol, robinin, lobetyolin, rutin, 3-O-β-D-glucopyranosyl platycodigenin and linoleic acid) were weighed and transferred into a 10-ml volumetric flask. Methanol was added to reach the volumetric mark, and the solution was shaken well, stored at 4 °C and used as a stock solution. Then, the appropriate amount of stock solution was transferred into a 5 ml volumetric flask, and methanol was added to reach the volumetric mark. The solutions were filtered using a 0.22 μm microporous membrane to obtain the standard solutions.

A total of 2.0 g PG powder was accurately weighed and transferred into a round bottom flask with 50 ml 50% methanol. The solution was mixed well, incubated for 0.5 h at room temperature, and ultrasonically treated for 30 min using an ultrasonic cleaning instrument (Jiangsu, China). The extracted solution was centrifuged at 14,000 rpm for 15 min at room temperature, and filtered using a 0.22 μm microporous membrane before qualitative analysis.

#### UPLC-Q-TOF-MS/MS Conditions

The chemical analysis was performed on a connected UPLC system (Nexera X2 LC-30A, Shimadzu Corp., Japan) -hybrid triple quadruple time-of-flight mass spectrometer (Triple TOF™ 5600^+^, AB Sciex, Forster City, CA, United States) with an electrospray ionization source (ESI). Acquity UPLC BEH C_18_ column (2.1 × 100 mm × 1.7 µm) was used to perform chromatographic separation with a flow rate of 0.25 ml/min at 40 °C. The linear gradient program used in this work was composed of a mobile phase system including solvent A (100% acetonitrile, v/v) and solvent B (0.01% formic acid in water, v/v) according to this detailed composition: solvent A at 5–23% for 10 min, 23–25% for 6 min, 25% for 4 min, 25–29% for 3 min, 29–95% for 7 min, 95–5% for 2.1 min, isocratic eluted at 5% for 2.9 min.

The settings of Q-TOF-MS/MS parameters were as follows: ion source gas 1 (GSI) and gas 2 (GS2) were both set at 50 psi, curtain gas (CUR) was set at 40 psi, ion spray voltage floating (ISVF) was set at 5500 V in the positive mode while 4500 V was set in the negative mode, ion source temperature (TEM) was set at 500 °C, collision energy (CE) was set at 60 V, collision energy spread (CES) was set at 15 V, declustering potential (DP) was set at 100 V, and nitrogen was used as a nebulizer and auxiliary gas. Samples were analyzed in both positive and negative ionization modes with a scanning mas-to-charge (m/z) range from 100 to 1,250. Data were collected in the information-dependent acquisition (IDA) mode and analyzed by PeakView®1.2 software (AB Sciex, Foster City, CA, United States).

#### Identification of the Ingredients

The chemical PG ingredients were collected from existing databases, such as SciFinder (https://scifinder.cas.org), the Traditional Chinese Medicine Systems Pharmacology database, and the Analysis Platform (TCMSP, http://lsp.nwu.edu.cn/tcmsp.php) database. Then, a PG ingredients database was established, containing the basic information, such as ingredient name and molecular formula. A total of 161 ingredients in PG were collected, and the specific information is listed in the Schedule 1. MS data were imported into PeakView^®^ 1.2 to perform the ingredient analysis. Chemicals were identified according to the reference standards, chromatographic elution behavior, mass fragment pattern, and mass spectral library (Natural Products HR-MS/MS Spectral Library, Version 1.0, AB Sciex, Forster City, United States).

### Predicted and Screened Potential Targets

The prediction targets of the active PG components were obtained using the following databases: Swiss Target Prediction (http://www.swisstargetprediction.ch/) (Xu et al., 2018), Pharmmapper (http://www.lilab-ecust.cn/pharmmapper/) (Shen et al., 2019), Pubchem (https://pubchem.ncbi.nlm.nih.gov/) (Xu et al., 2018) and TCMSP (http://lsp.nwu.edu.cn/tcmsp.php) (Ye et al., 2020). The protein name was standardized using Uniprot (http://www.uniprot.org/), and the library of the targets of the active components was obtained. The known disease targets were searched in the databases of GeneCards (https://www.genecards.org/) (Ye et al., 2020) and DisGenet (http://www.disgenet.org/) (Gao et al., 2020) using “Lung cancer” as the keyword, and the LC-target database was obtained.

### Protein-Protein Interaction (PPI) Network Construction

R software was used to intersect PG related targets and LC related targets, and the overlapping targets were uploaded into STRING 11.0 (https://string-db.org/). The protein type was set to “*Homo sapiens*” and the minimum interaction score was 0.4. The PPI network diagram was obtained, imported into Cytoscape v3.7.1 software, and CentiScape was used to calculate the degree centrality (DC) to filter PPI network core targets.

### Gene Ontology (GO) and Kyoto Encyclopedia of Genes and Genomes (KEGG) Pathway Enrichment Analysis

Metascape (http://www.metascape.org/) is a gene annotation tool that integrates many authoritative databases such as GO, KEGG, UniProt and DrugBank. It allows the completion of pathway enrichment analysis and biological process annotation and performs gene-related protein network analysis and drug analysis, providing comprehensive and detailed information regarding each gene (Zhou et al., 2019). Metascape perfectly fills the gap of DAVID while maintaining its advantages, and its data is frequently updated (the last update was August 14, 2019), greatly guaranteeing the timeliness and credibility of the data.

The gene symbols of the core targets were introduced into Metascape, “*Homo sapiens*” was selected to perform the enrichment analysis, which was annotated and analyzed using the KEGG database (https://www.kegg.jp/) and PathwayBuilderTool_2.0 software to further explain the role of the core targets in gene function and signaling pathway.

### D-I-T-P-D Network Construction

The files related to “drug-core ingredient,” “core ingredient-core target,” “pathway-core target,” and “disease-core target” were established, and the D-I-T-P-D network was constructed using Cytoscape v 3.7.1 to explain the multi-effect synergistic mechanism of PG.

### Molecular Docking Evaluation

Discovery Studio 2016 software (DS) is a commonly used molecular modeling and simulation software widely used in the field of drug design and optimization, protein structure and function (Zhang and Mao, 2018). The PDB format of the core target is obtained in the Uniprot database, and the X-ray crystal structure of the core target is downloaded from the RCSB database (https://www.rcsb.org/). Then the molecular docking function of DS software is used to perform component-target molecular docking in the LibDock module.

## Results

### Composition Analysis and Identification

**FIGURE 2:**
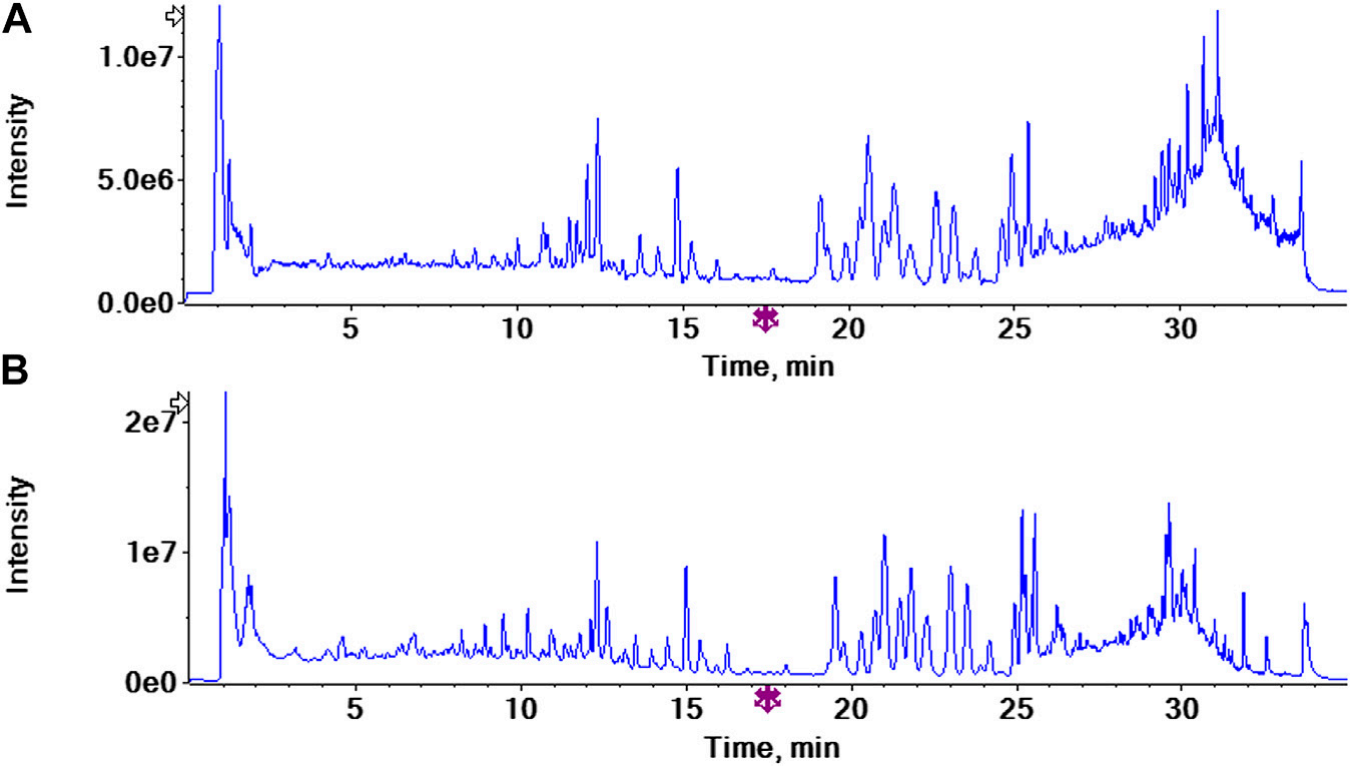
The total ion chromatograms (TICs) of *Platycodon grandiflorum* by UPLC/Q-TOF-MS/MS **(A)** TIC of *Platycodon grandiflorum* in positive ion mode **(B)**TIC of *Platycodon grandiflorum* in negative ion mode.

The typical total ion chromatogram of the non-volatile components extracted from PG, shown in Figure 2, of positive and negative ions was analyzed by Peakview ^®^1.2, and the composition was screened by “XIC manager”. The structural formula of the target compound was matched with the secondary fragment ion, and the compounds were further identified according to the matching degree and the law of ion bond breaking. The compounds with a matching degree greater than 80% and in accordance with the law of bond breaking were considered. According to the reference (Wang et al., 2017a; 2017b) and the data of the control quality spectrum, 47 chemical constituents were identified in the alcohol extract of PG (Table 1). Among them, deapio-platycodin D, platycodin D, polygonatoside C1, adenosine, ferulic acid, apigenin, luteolin, chlorogenic acid, caffeic acid, kaempferol, robinin, lobetyolin, rutin, 3-O-β-D-glucopyranosyl platycodigenin and linoleic acid were compared with the reference standards. Among these 47, 24 were triterpenoid saponins, three were steroidal saponins, six were flavonoids, three were phenolic acids, four were organic acids and seven were other components.

**TABLE 1:** Information on chemical ingredients in *Platycodon grandiflorum*.

NO	*t*_*R*_/min	Molecular Formula	ESI-MS	Error (ppm)	Fragment ions m/s	Identity	Sort
1	11.99	C_42_H_68_O_16_	**827.4435** [M-H]^−^	−2	707.3943,**665.3866**,647.3830,503.3403,441.2817	platycodon A	triterpene saponins
2	11.99	C_42_H_68_O_16_	**827.4435** [M-H]^−^	−2	707.3943,**665.3866**,647.3830,503.3403,441.2817	platycosaponin A	triterpene saponins
3	13.04	C_41_H_66_O_15_	797.4329 [M-H]^−^	−2.1	**665.3987**,647.3789,**635.3509**,617.3651,485.3600,469.1602	platycodon B	triterpene saponins
4	14.09	C_52_H_84_O_23_	**1075.5331** [M-H]^−^	−2.7	925.4664,**665.3873**,647.3716,485.3034,441.3356,337.1133	platycoside J	triterpene saponins
5	14.55	C_36_H_54_O_12_	677.3543 [M-H]^−^	−3.3	615.3600,515.2936,453.3113	platycoside M1	triterpene saponins
6^a^	19.51	C_52_H_84_O_24_	1091.5280 [M-H]^−^	−2.5	**681.3853**,663.3734,619.3120,**635.3797**,519.3297,501.3211,471.3097,457.3321,337.1140	deapio-platycodin D	triterpene saponins
7	19.99	C_58_H_94_O_28_	1237.5859 [M-H]^−^	−2.1	**827.4311**,503.3298,483.1761,471.3029,483.1761	platycoside H	triterpene saponins
8	20.73	C_57_H_92_O_28_	1223.5702 [M-H]^−^	−3.7	663.3602,**635.3793**,619.3788,501.3227,471.3065,457.3240,441.3025	2′-*O*-polygalacin D	triterpene saponins
9^a^	20.73	C_57_H_92_O_28_	1223.5702 [M-H]^−^	−3.7	**1133.5309,681.3789**,541.1729,501.3189,469.1518,451.3297,409.1329	platycodin D	triterpene saponins
10	20.73	C_57_H_92_O_28_	1223.5702 [M-H]^−^	−3.7	**1133.5309,681.3789**,541.1729,501.3189,469.1518,451.3297,409.1329	3′*-α-O*-polygalacin D	triterpene saponins
11	21.27	C_52_H_82_O_25_	1105.5072 [M-H]^−^	−2.8	**1075.4941**,895.4301,485.2880	platyconic acid C	triterpene saponins
12	21.95	C_57_H_92_O_27_	**1207.5753** [M-H]^−^	−3.2	**665.3907**,541.1756,469.1544	polygalacin D	triterpene saponins
13	22.74	C_57_H_90_O_29_	1237.5495 [M-H]^−^	−2.2	**1207.5346**,1027.4720	platycodin J	triterpene saponins
14	22.74	C_57_H_90_O_29_	1237.5495 [M-H]^−^	−2.2	**1207.5346**,1027.4720	platyconic acid A	triterpene saponins
15	23.73	C_54_H_86_O_25_	**1133.5385** [M-H]^−^	−2.4	1091.5274,723.3920,691.3822,663.3695,501.3229,337.1144	platycoside B	triterpene saponins
16	23.73	C_54_H_86_O_25_	**1133.5385** [M-H]^−^	−2.4	1091.5274,723.3920,691.3822,663.3695,501.3229,337.1144	platycoside C	triterpene saponins
17	24.79	C_57_H_90_O_28_	1221.5546 [M-H]^−^	−2.5	469.1542	16-*OXO*-platycodin D	triterpene saponins
18	25.11	C_54_H_84_O_26_	1147.5178 [M-H]^−^	−2.2	1117.5032,937.1142,485.2896	platyconic acid D	triterpene saponins
19^a^	25.84	C_36_H_58_O_12_	**681.3856** [M-H]^−^	−4.3	**635.3761**,471.3072,457.3307,379.2971	3-*O-β-D*-glucopyranosyl platycodigenin	triterpene saponins
20	26.14	C_36_H_58_O_11_	**665.3906** [M-H]^−^	−3.4	619.3991,503.3352,441.3325,101.0291	3*-O-β-D*-glucopyranosyl polygalacic acid	triterpene saponins
21	26.92	C_30_H_46_O_8_	533.3120 [M-H]^−^	−3.6	469.2915	platycogenic acid B	triterpene saponins
22	26.92	C_30_H_46_O_8_	533.3120 [M-H]^−^	−3.6	485.2892,469.2915,441.3001,377.2838	platycogenic acid A	triterpene saponins
23	27.81	C_35_H_56_O_10_	**635.3801** [M-H]^−^	−4	473.3247,443.3125,425.3030,423.2874,379.2631,217.1586,119.0367	platycodonoids B	triterpene saponins
24	28.61	C_18_H_34_O_4_	313.2382 [M-H]^−^	−0.8	212.9942,210.1123,199.0968,171.1025,165.0937,155.1079,137.0994,127.1165	dibutyl sebacate	triterpene saponins
25^a^	13.02	C_44_H_70_O_17_	869.4540 [M-H]^−^	−0.4	809.3746,707.3538	polygonatoside C1	steroidal saponins
26	25.88	C_51_H_80_O_24_	1075.4967 [M-H]^−^	−1.2	**665.4041,503.2971**,485.1902,409.1317	cyrtonemoside A	steroidal saponins
27	25.88	C_51_H_80_O_24_	1075.4967 [M-H]^−^	−1.2	**665.3867**,619.3991,503.3352,441.3325,101.0291	(25*S*) spirostan-5-en-12-one-3-*O-β-D*-glucopyranosyl- (1→2) -*O*-[*β-D-g*lucopyranosyl- (1→3) ]-*β-D-*glucopyranosyl-(1→4) -*β-D*-galactopyranoside	steroidal saponins
28^a^	7.32	C_27_H_30_O_16_	611.1607 [M+H]^+^	−0.8	**449.0946**,315.0935,287.0541,**269.0503**	rutin	flavonoids
29	9.54	C_21_H_20_O_11_	**449.1078** [M+H]^+^	−3.3	287.0546,**269.0432**,203.0322,135.0441,161.0245	luteolin-7-*O*-glucoside	flavonoids
30^a^	11.65	C_33_H_40_O_19_	739.2091 [M-H]^−^	−3	221.0714,179.0589,161.0497	robinin	flavonoids
31^a^	14.61	C_15_H_10_O_6_	285.0405 [M-H]^−^	−0.6	267.0299,201.0132,175.0421,151.0087,133.0303,107.0166	luteolin	flavonoids
32^a^	14.65	C_15_H_10_O_6_	285.0405 [M-H]^−^	−0.3	267.0299,217.0512,175.0421,151.0087,133.0308,107.0166	kaempferol	flavonoids
33^a^	18.55	C_15_H_10_O_5_	**269.0456** [M-H]^−^	−0.8	159.0449,117.0384,115.0085,107.0149	apigenin	flavonoids
34^a^	5.10	C_16_H_18_O_9_	353.0878 [M-H]^−^	−3.6	191.0569,189.0419,173.0479,171.0325,155.0334,135.0471,107.0499	chlorogenic acid	phenolic acid
35^a^	5.97	C_9_H_8_O_4_	179.0350 [M-H]^−^	3.9	135.0502,133.0340,117.0441,107.0578	caffeic acid	phenolic acid
36^a^	10.00	C_10_H_10_O_4_	193.0506 [M-H]^−^	0.9	133.0344	ferulic acid	phenolic acid
37	7.03	C_12_H_14_O_5_	239.0914 [M+H]^+^	−3	147.0456,119.0505	1-*O*-*p*-coumaroylglycerol	organic acid
38	26.23	C_18_H_34_O_5_	329.2334 [M-H]^−^	−0.9	311.2160,293.2189,211.1344,185.1110,171.1036,129.0907	sanleng acid	organic acid
39	30.76	C_19_H_32_O_2_	293.2475 [M+H]+	−3.8	135.1172,115.0549,109.1014,105.0720	methyl linolenate	organic acid
40^a^	31.87	C_18_H_32_O_2_	279.2330 [M-H]^−^	0.8	261.2150	linoleic acid	organic acid
41^a^	1.35	C_10_H_13_N_5_O_4_	268.1040 [M+H]+	−0.5	136.0621,119.0354	adenosine	others
42	30.13	C_28_H_42_N_4_O_6_	529.3008 [M-H]^-^	−4.5	279.2337,246.0625	kukoamine A	others
43	30.28	C_20_H_34_O_2_	307.2632 [M+H]^+^	−3.2	217.1018,153.0783,131.0854,119.0864,107.0861	(9*Z*,12*Z*,15*Z*)-ethyl octadeca-9,12,15-trienoate	others
44	31.78	C_18_H_35_NO	282.2792 [M+H]^+^	−2.9	114.0931	oleamide	others
45^a^	10.72	C_20_H_28_O_8_	397.1857 [M+H]^+^	−2	153.0670,127.0592,115.0598,105.0346	lobetyolin	others
46	8.22	C_29_H_42_O_18_	677.2298 [M-H]^−^	−1.4	261.1000,161.0471,153.0584,143.0577,125.0275	tangshenoside Ⅰ	others
47	4.58	C_11_H_12_N_2_O_2_	203.0830 [M-H]^−^	1.8	142.0664,130.0694,116.0542	tryptophan	others

^a^ Structures confirmed by comparison with reference standards

### Analysis of the Cracking Law of the Components

#### Triterpene Saponins

**FIGURE 3:**
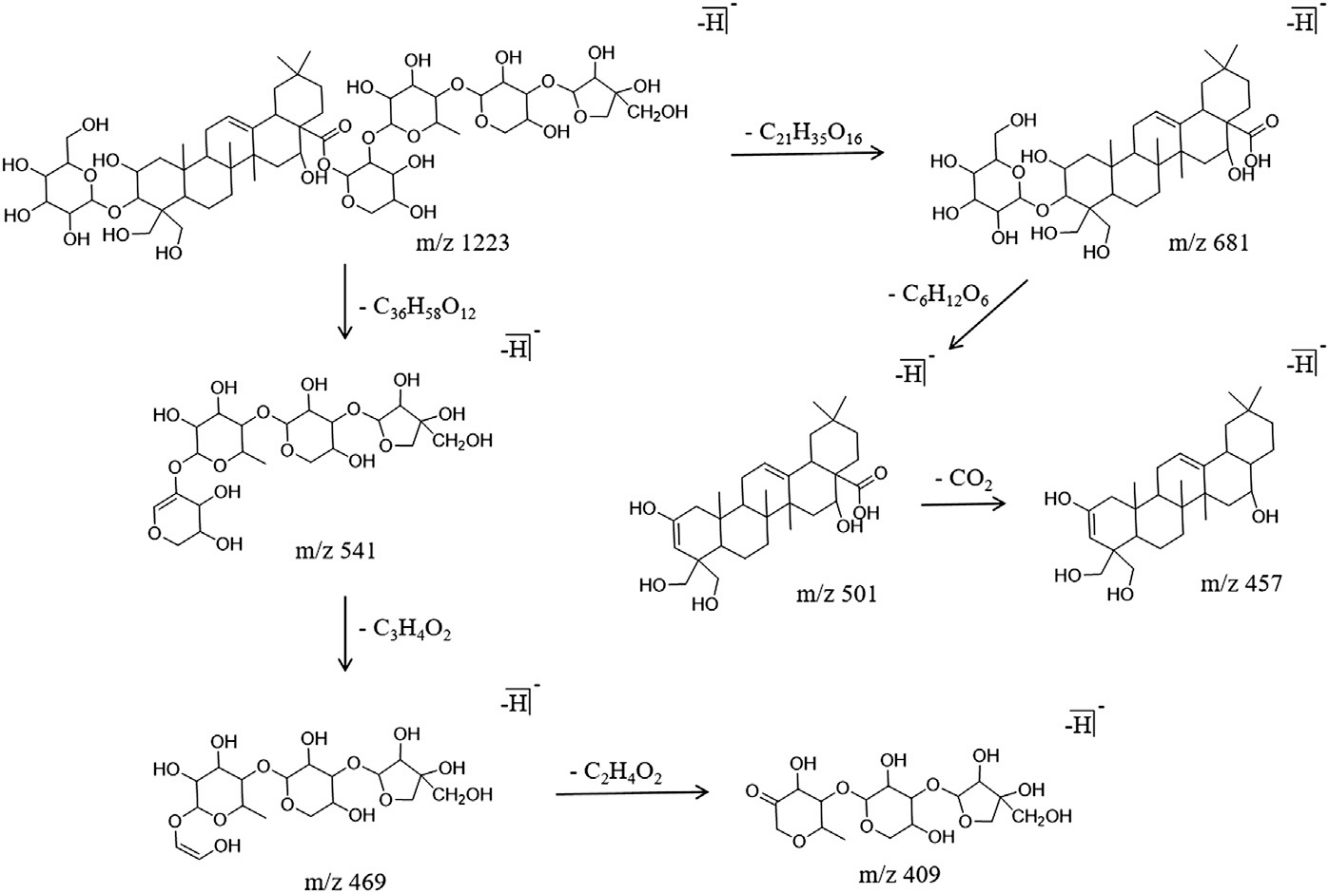
Cracking pathway of triterpenoid saponins.

Triterpenoid saponins are the largest number of components and the main components identified from PG. The main approach to cleave triterpenoid saponins is a continuous intramolecular deglycosylation and interchain cleavage to obtain the characteristic fragment ions. In this experiment, 24 triterpenoid saponins, including platycodin D, polygalacin D and deapio-platycodin D were identified from PG. Using platycodin D as an example for the analysis, they all behave similarly by producing a molecular ion peak m/z 1,223.5702 in the anion mode, and the retention time was 20.73 min. The glycosidic bond was broken under a high energy bombardment, the fragment ions m/z 681.3789 [M-H]^−^, m/z 541.1729 [M-H]^−^, m/z 681.3789 [M-H]^−^ were further removed, and the fragment ion m/z 501.3189 [M-H]^−^ was obtained. In addition, the interchain cleavage produced the fragment ions m/z 469.1518 [M-H]^−^ and m/z 409.1329 [M-H]^−^. The specific cracking pathway of the triterpenoid saponins is shown in Figure 3.

#### Steroidal Saponins

**FIGURE 4:**
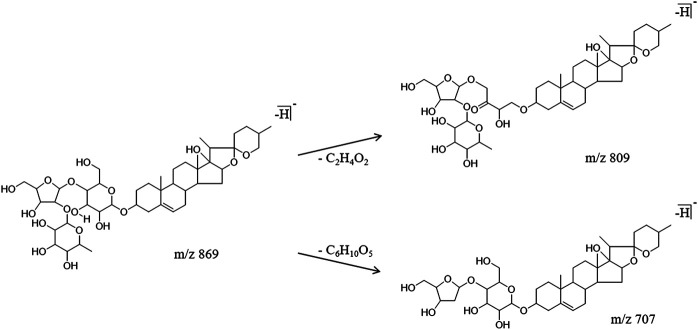
Cracking pathway of steroidal saponins.

Three steroidal saponins were identified and their cleavage rules were similar to the ones to cleave triterpenoid saponins. The characteristic fragment ions were obtained by the cleavage of their sugar chains. Using polygonatoside C1 as an example for the analysis, the molecular ion peak m/z 869.4540 was produced in the negative ion mode, and the retention time was 13.02 min. The cleavage of the sugar chain produced the fragment ions m/z 809.3746 [M-H]^−^ and m/z 707.3538 [M-H]^−^. The specific cracking pathway of the steroidal saponins is shown in Figure 4.

#### Flavonoids

**FIGURE 5:**
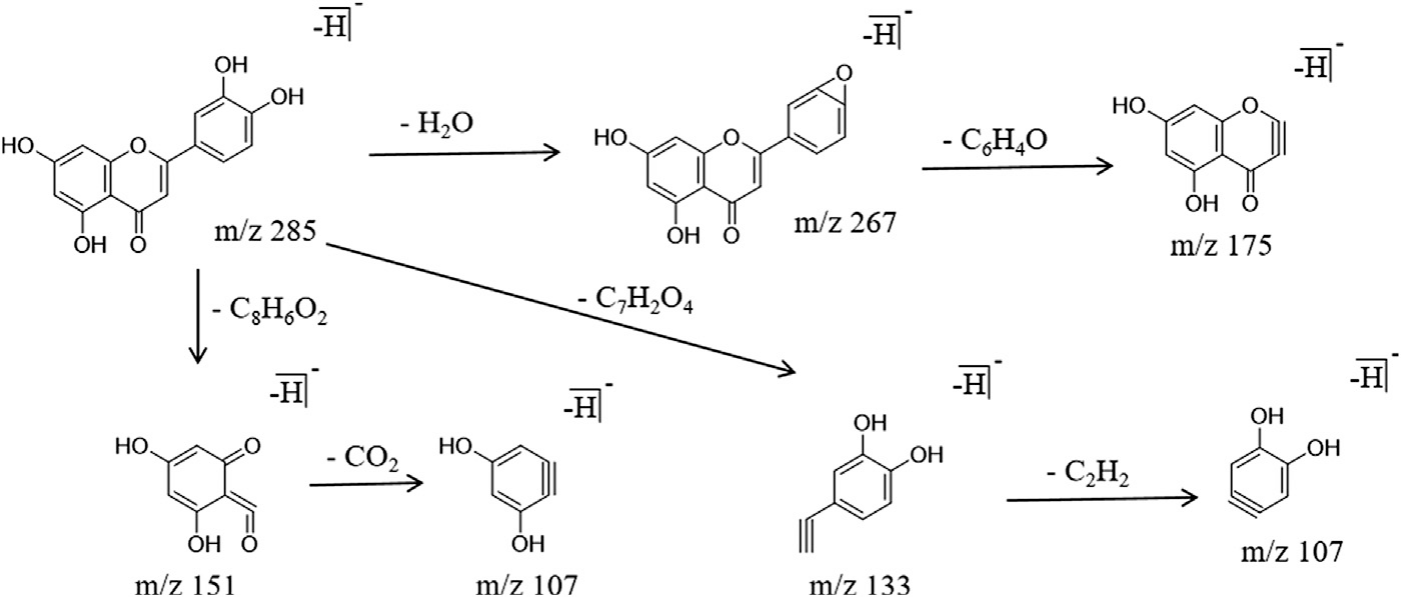
Cracking pathway of flavonoids.

It is already known that flavonoids are the main components in PG (Wang et al., 2017b; Deng et al., 2020). The main components identified were flavonoids and flavonoid glycosides, and the characteristic cleavage mode was the R-DA cleavage, while glycosides followed the glycosyl cleavage. Using luteolin as an example for the analysis, the molecular ion peak m/z 285.0405 was produced in the negative ion mode, and the retention time was 14.61 min the characteristic fragment ions m/z 151.0087 [M-H]^−^, m/z 133.0303 [M-H]^−^, and m/z 107.0166 [M-H]^−^ were obtained, respectively, by R-DA cleavage at two different positions. In addition, the fragment ion m/z 267.0299 [M-H]^−^ was obtained by removing one molecule of H_2_O, and the fragment ion m/z 175.0421 [M-H]^−^ was also obtained by alpha cracking. The specific cracking pathway of the flavonoids is shown in Figure 5.

#### Phenolic Acids

**FIGURE 6:**
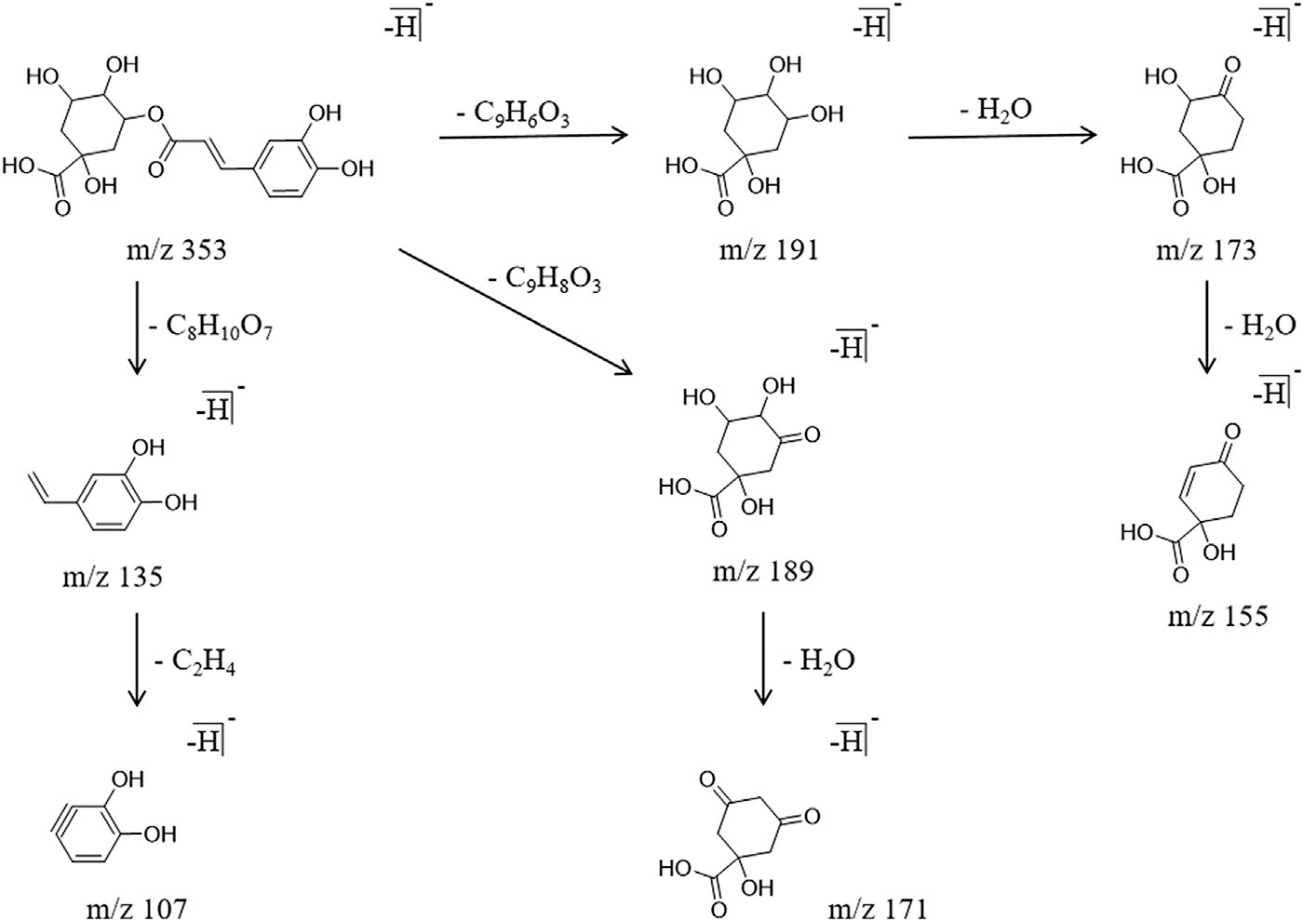
Cracking pathway of phenolic acids.

Three phenolic acid components were identified from PG. The main cracking methods were alpha cracking, Michael rearrangement cracking, and neutral H_2_O molecule detachment. Using the chlorogenic acid as an example for the analysis, it generated a molecular ion peak m/z 353.0878 in the negative ion mode, with a retention time of 5.10 min. There are three main cracking methods: first, the alpha cleavage produced the fragment ion m/z 191.0569 [M-H]^−^, and two molecules of H_2_O were removed to obtain the fragment ion m/z 173.0479 [M-H]^-^ and m/z 155.0334 [M-H]^−^. Second, the cleavage by Michael rearrangement produced the fragment ion m/z 189.0419 [M-H]^−^, and then a molecule of H_2_O was removed to obtain the fragment ion m/z 171.0325 [M-H]^−^. Third, the alpha fragmentation produced the fragment ion m/z 135.0471 [M-H]^−^ and m/z 107.0499 [M-H]^−^. The specific cracking pathway of the phenolic acids is shown in Figure 6.

#### Organic Acids

**FIGURE 7:**
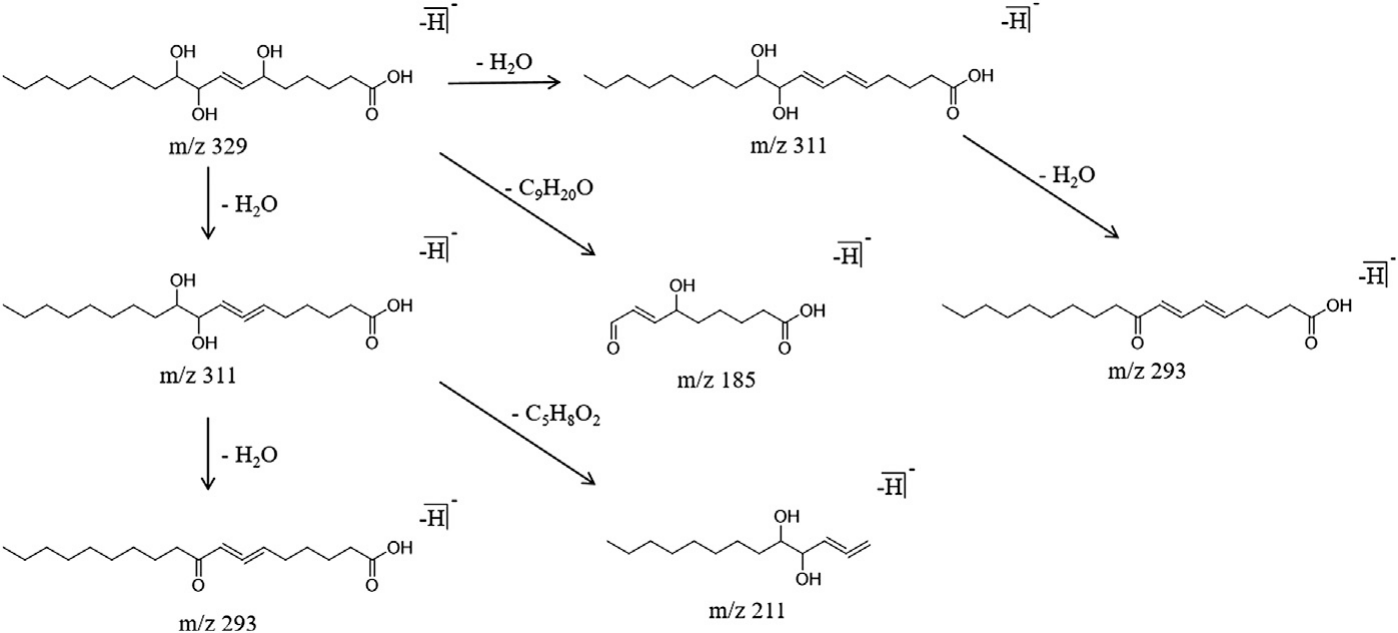
Cracking pathway of organic acids.

The organic acid components identified in this work were mainly long-chain carboxylic acids with multiple unsaturated bonds. The characteristic cleavage method involved the Michael rearrangement cracking, alpha cracking and neutral molecule removal. Using the sanleng acid as an example for the analysis, it generated a molecular ion peak m/z 329.2334 in the negative ion mode with a retention time of 26.23 min. Michel rearrangement fragmentation produced the characteristic fragment ion m/z 185.1110 [M-H]^-^. The fragment ion m/z 311.2160 [M-H]^-^ was obtained by removing one molecule of H_2_O. Another molecule of H_2_O was removed from m/z 311.2160 [M-H]^−^ to obtain the fragment ion m/z 293.2189 [M-H]^-^; in addition, m/z 311.2160 [M-H]^−^ underwent alpha cracking to obtain the fragment ion m/z 211.1344 [M-H]^−^. The specific cracking pathway of the organic acids is shown in Figure 7.

### Screening of the Active Ingredients

The absorption-distribution-metabolism-excretion-toxicity (ADMET) properties of the chemical constituents were predicted by DS, and apigenin, caffeic acid, kaempferol, linoleic acid, methyl linolenic acid, and ferulic acid were selected. Although some components do not meet the DS screening criteria, they are still considered as active components in order to select the active components of PG more comprehensively. For example, luteolin and robinin were obtained from TCMSP, with oral bioavailability greater than 30% and drug-likeness greater than 0.18 despite they did not meet the DS screening criteria, so they are considered as active ingredients. Recent studies showed that platycodin D is one of the main components of PG, and has a certain preventive and therapeutic effect on LC (Zhao et al., 2015; Zhang, 2016b; Deng et al., 2020), thus platycodin D is also retained as the active ingredient. To sum up, a total of nine ingredients were selected as the active components of PG, and their detailed characteristics are listed in Table 2.

**TABLE 2:** Information on the 9 active ingredients in *Platycodon grandiflorum.*

Note	CAS	Molecular formula	Molecular weight	Compound
S1	1135-24-6	C_10_H_10_O_4_	194.058	ferulic acid
S2	520-36-5	C_15_H_10_O_5_	270.053	apigenin
S3	520-18-3	C_15_H_10_O_6_	286.048	kaempferol
S4	331-39-5	C_9_H_8_O_4_	180.042	caffeic acid
S5	7361-80-0	C_19_H_32_O_2_	292.240	methyl linolenate
S6	60-33-3	C_18_H_32_O_2_	280.240	linoleic acid
S7	491-70-3	C_15_H_10_O_6_	286.048	luteolin
S8	301-19-9	C_33_H_40_O_19_	740.216	robinin
S9	58479-68-8	C_57_H_92_O_28_	1224.578	platycodin D

### PPI Network Analysis

A total of 545 targets of the selected nine active components in PG were obtained using the Swiss Target Prediction, Pubchem, TCMSP and pharmmapper databases. A total of 2664 LC related targets were collected using GeneCards and DisGenet databases using “Lung cancer” as the keyword (Schedules 2,3).

**FIGURE 8:**
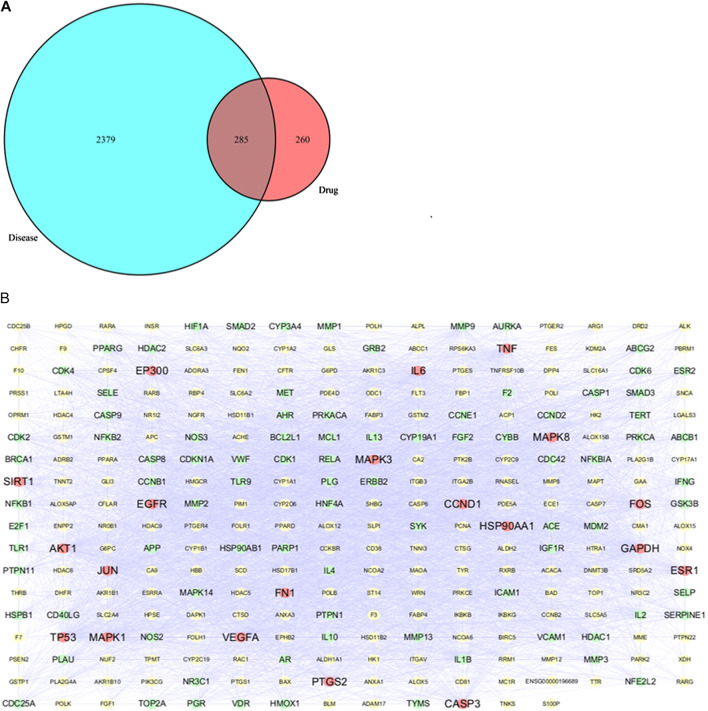
**(A)** Venny diagram of related targets of drug and disease **(B)** PPI network of 285 overlapping targets between drug and disease.

The Venn diagram of drug-disease overlapping targets was obtained using the R software to intersect PG related targets and LC related targets (Figure 8A), revealing the existence of 285 common targets (Schedule 4). The interactive PPI network of common targets was obtained by introducing the 285 drug-disease common targets into STRING and the obtained network was imported into the Cytoscape software for visualization (Figure 8B). No interaction was found between the target S100P and other targets, but the other 284 common targets had an interaction. The average value of DC calculated by CentiScape was 39.775, and 108 potential targets with DC values greater than 39.775 were selected. The higher the DC value, the more important the role in the network. In this study, the targets with the top 20 DC values were selected as the core targets. The red dot in Figure 8B represents the core target, the light green dot represents the potential target, the yellow dot represents the interactive target, and the connection between the nodes represents the interaction between the two proteins.

### GO and KEGG Pathway Enrichment Analysis

**FIGURE 9:**
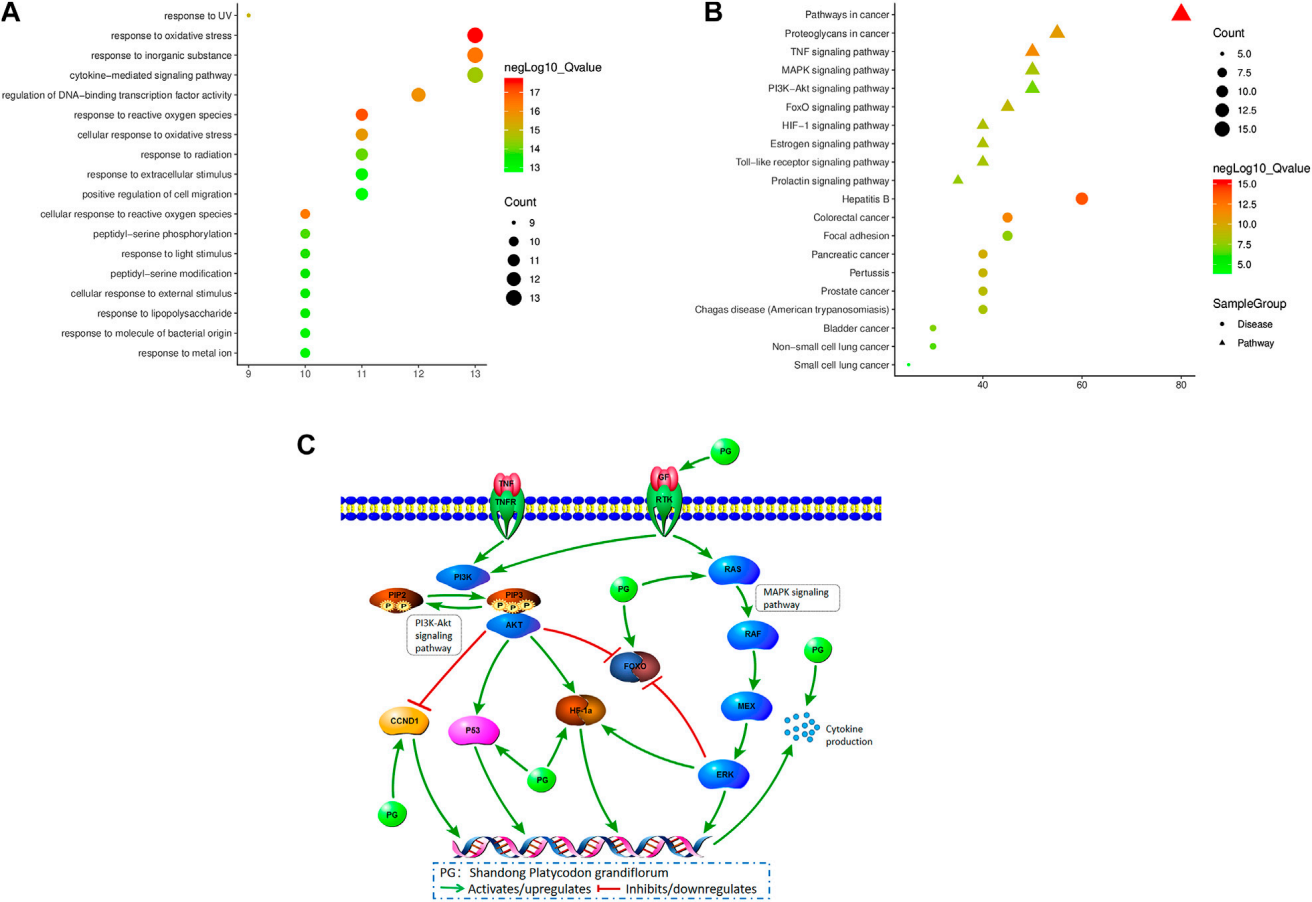
**(A)** Bubble chart of GO function enrichment of core targets **(B)** Bubble chart of KEGG enrichment of core targets **(C)** Potential mechanisms of *Platycodon grandiflorum* treatment for lung cancer.

The pathway enrichment analysis of the 20 core targets was carried out using Metascape. The enrichment results were selected under the conditions of *p*< 0.01, minimum count 3, and enrichment factor>1.5. A total of 849 GO biological functions and 116 KEGG enrichment items were obtained. The GO functions related to the treatment of LC included oxidative stress response (GO:0006979), active regulation of cell migration (GO:0030335), regulation of DNA binding transcription factor activity (GO:0051090), and regulation of cytokine-mediated signaling pathway (GO:0019221) (Figure 9A), mostly related to apoptosis, oxidative stress and energy metabolism. The 20 core targets were closely related to cancer pathway (hsa05200), TNF signaling pathway (hsa04668), MAPK signaling pathway (hsa04010) and P13K-AKT signaling pathway (hsa04151), and are related to diseases such as hepatitis B, colorectal cancer, non-small cell LC and small cell LC (Figure 9B). The first 20 representative signaling pathways are listed in Table 3, which might represent the key pathways in the treatment of LC. It is also suggested that PG can be used in the treatment of LC. The top 10 pathways were determined in the KEGG database and annotated using the PathwayBuilderTool_2.0 software to integrate the potential pathways of PG in the treatment of LC (Figure 9C). This analysis provided a new research method for the limitations in treating LC.

**TABLE 3:** Top 20 clusters with their representative KEGG enrichment pathways.

ID	Term	Count	LogP	Gene
hsa05200	Pathways in cancer	16	−25.05	AKT1、CCND1、CASP3、EGFR、EP300、FN1、FOS、HSP90AA1、IL6、JUN、MAPK1、MAPK3、MAPK8、PTGS2、TP53、VEGFA
hsa05205	Proteoglycans in cancer	11	−17.75	AKT1、CCND1、CASP3、EGFR、ESR1、FN1、MAPK1、MAPK3、TNF、TP53、VEGFA
hsa04668	TNF signaling pathway	10	−18.43	AKT1、CASP3、FOS、IL6、JUN、MAPK1、MAPK3、MAPK8、PTGS2、TNF
hsa04010	MAPK signaling pathway	10	−14.62	AKT1、CASP3、EGFR、FOS、JUN、MAPK1、MAPK3、MAPK8、TNF、TP53
hsa04151	PI3K-Akt signaling pathway	10	−13.33	AKT1、CCND1、EGFR、FN1、HSP90AA1、IL6、MAPK1、MAPK3、TP53、VEGFA
hsa04068	FoxO signaling pathway	9	−15.28	AKT1、CCND1、EGFR、EP300、IL6、MAPK1、MAPK3、MAPK8、SIRT1
hsa04915	Estrogen signaling pathway	8	−14.18	AKT1、EGFR、ESR1、FOS、HSP90AA1、JUN、MAPK1、MAPK3
hsa04066	HIF-1 signaling pathway	8	−14.07	AKT1、EGFR、EP300、GAPDH、IL6、MAPK1、MAPK3、VEGFA
hsa04620	Toll-like receptor signaling pathway	8	−13.97	AKT1、FOS、IL6、JUN、MAPK1、MAPK3、MAPK8、TNF
hsa04917	Prolactin signaling pathway	7	−13.02	AKT1、CCND1、ESR1、FOS、MAPK1、MAPK3、MAPK8
hsa04919	Thyroid hormone signaling pathway	7	−11.44	AKT1、CCND1、EP300、ESR1、MAPK1、MAPK3、TP53
hsa04921	Oxytocin signaling pathway	7	−10.61	CCND1、EGFR、FOS、JUN、MAPK1、MAPK3、PTGS2
hsa04621	NOD-like receptor signaling pathway	7	−10.27	HSP90AA1、IL6、JUN、MAPK1、MAPK3、MAPK8、TNF
hsa04024	cAMP signaling pathway	7	−9.80	AKT1、EP300、FOS、JUN、MAPK1、MAPK3、MAPK8
hsa04012	ErbB signaling pathway	6	−10.20	AKT1、EGFR、JUN、MAPK1、MAPK3、MAPK8
hsa04660	T cell receptor signaling pathway	6	−9.72	AKT1、FOS、JUN、MAPK1、MAPK3、TNF
hsa04071	Sphingolipid signaling pathway	6	−9.36	AKT1、MAPK1、MAPK3、MAPK8、TNF、TP53
hsa04722	Neurotrophin signaling pathway	6	−9.34	AKT1、JUN、MAPK1、MAPK3、MAPK8、TP53
hsa04014	Ras signaling pathway	6	−7.65	AKT1、EGFR、MAPK1、MAPK3、MAPK8、VEGFA
hsa04370	VEGF signaling pathway	5	−8.96	AKT1、MAPK1、MAPK3、PTGS2、VEGFA

### D-I-T-P-D Network Analysis

**FIGURE 10:**
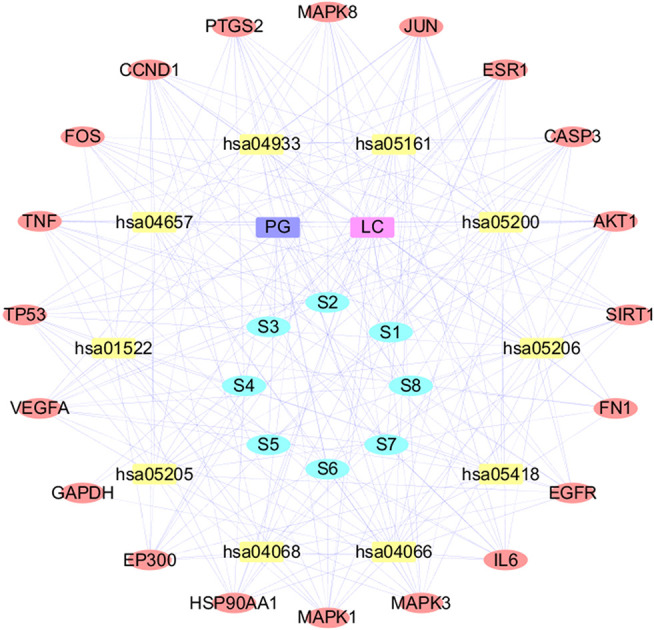
The Drug-Ingredients-Targets-Pathway-Disease Network. S1: ferulic acid; S2: apigenin; S3: kaempferol; S4: caffeic acid; S5: methyl linolenate; S6: linoleic acid; S7: luteolin; S8: robinin; PG: *Platycodon grandiflorum*; LC: lung cancer.

In order to explain the complex mechanism of multi-ingredient-multi-target-multi-pathway of PG in the treatment of LC, this study selected the top 10 pathways and used Ctyoscape v 3.7.1 to construct a D-I-T-P-D network, as shown in Figure 10. In this network, the purple node represents the drug, the green node represents the core ingredient of PG, the yellow node represents the core target of PG in the treatment of LC, the red node represents the pathway, the blue node represents the disease, and the edge represents thee interaction among nodes. The network consists of 40 nodes (1 drug, eight core components, 20 core targets, 10 pathways and one disease), with a total of 277 edges, which fully showed that the targets of the core ingredients of PG were distributed in different pathways, and the ingredients coordinated with each other in the body.

### Subsistence Analysis

**FIGURE 11:**
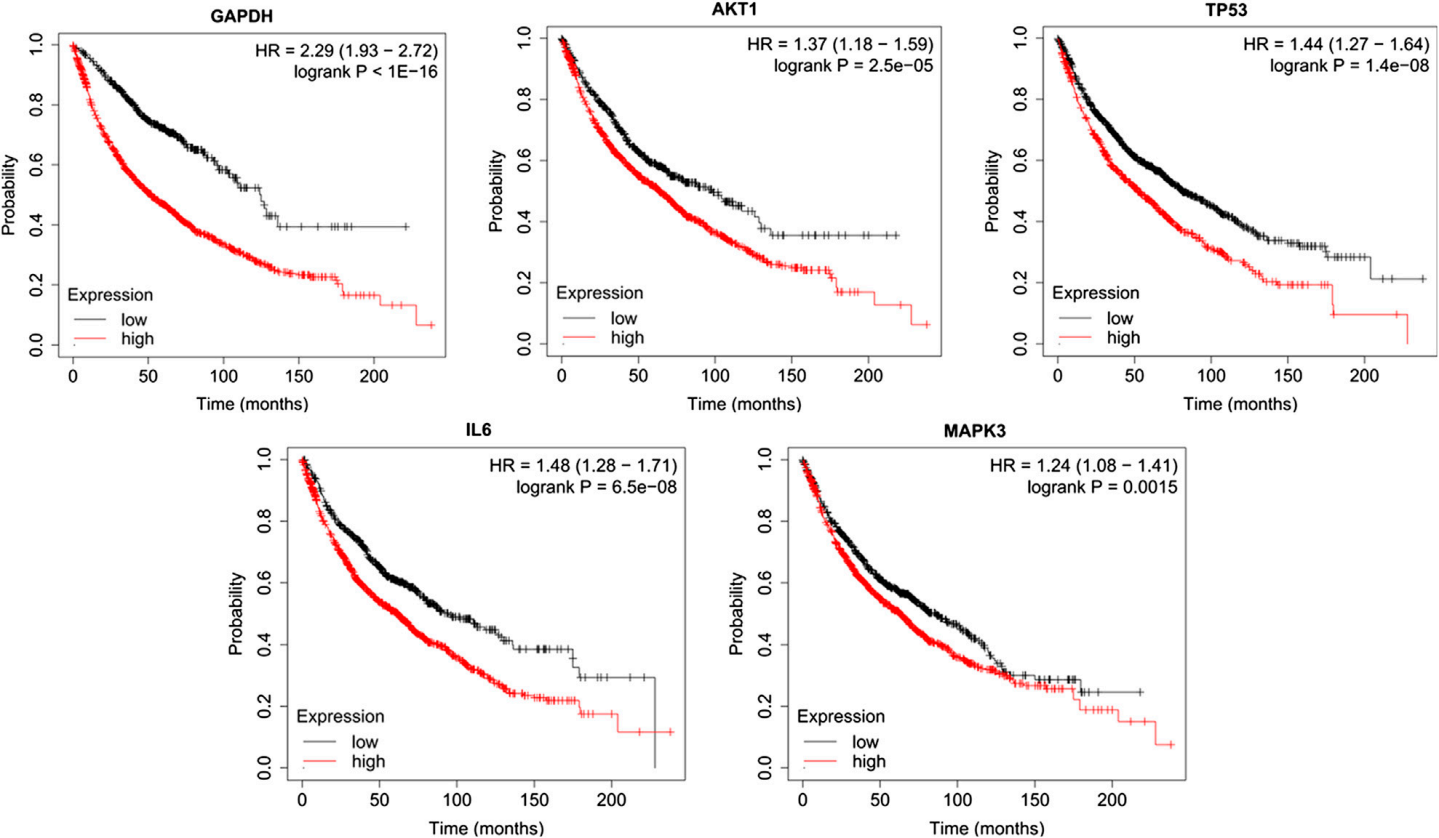
Survival curve of the relationship between high and low expression and overall survival of patients.

The relationship between the top five core targets in the PPI network and the prognosis of LC patients was analyzed using the Kaplan-Meier Plotter database. The results revealed that the overall survival of LC patients with high expression of GAPDG, AKT1, TP53, IL6 and MPKA3 was significantly lower than that in patients with their low expression (*p* < 0.01) (Figure 11).

### Molecular Docking Verification Results

**FIGURE 12:**
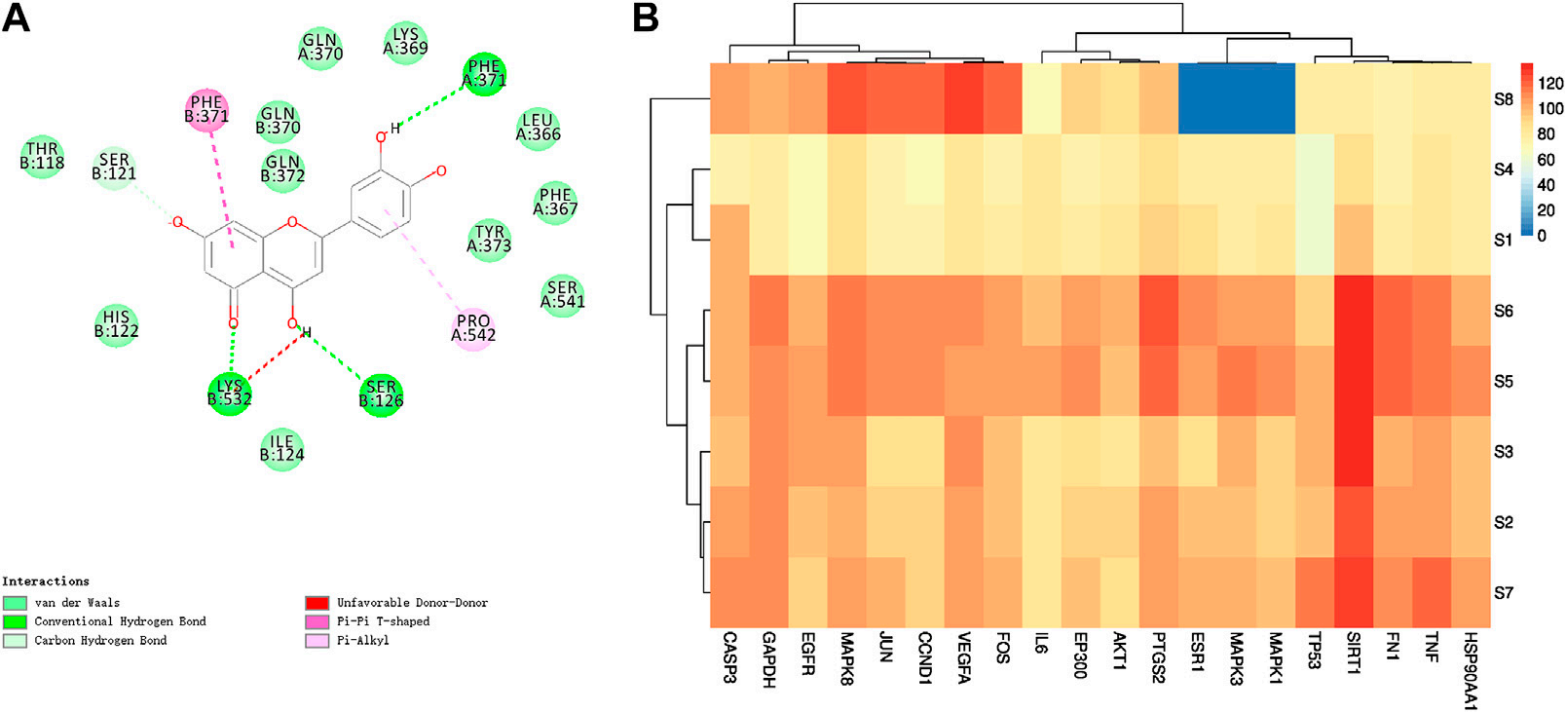
**(A)** 2D graph of luteolin and PTGS2 molecular docking results **(B)** Heatmap of molecular docking scores of core ingredients and core targets. S1: ferulic acid; S2: apigenin; S3: kaempferol; S4: caffeic acid; S5: methyl linolenate; S6: linoleic acid; S7: luteolin; S8: robinin.

The analysis of the D-I-T-P-D network showed that each core component interacted with multiple targets. Thus, the DS software was used to dock the core components with the core target molecules in order to verify the interaction among them. It is generally accepted that the higher the score of the ligand binding to the receptor, the greater the possibility of interaction. The docking results of luteolin and protein PTGS2 docking are shown in Figure 12A, which might bond by hydrogen bonds, van der Waals forces and other forces. The docking score was sorted out, the highest score was obtained (Schedule 5), and then ImageGP was used for visual processing (Figure 12B). The redder the color, the better the affinity of the component to the target. The relationship between the above-mentioned core components and core targets was consistent according to the analysis of the docking scoring results.

The cluster analysis revealed that GAPDH, VEGFA, EGFR, JUN, CCND1, MAPK8, FOS and CASP3 could be included into one group, while EP300, AKT1, HSP90AA1, MAPK3, TNF, SIRT1, ESR1, PTGS2, TP53, FN1, MAPK1 and IL6 could be included in one category. SIRT1, PTGS2, CASP3, VEGFA, MAPK8 and GAPDH were the targets with strong binding ability according to the molecular docking. Linoleic acid, methyl linolenate, luteolin, apigenin and kaempferol were the core components with strong binding ability according to the molecular docking, and the docking scores of these five components with SIRT1 were greater than 120, followed by methyl linolenate, kaempferol, linoleic acid, luteolin and apigenin. This result suggested that these five components have a good binding activity with SIRT1 and might play a key role in the treatment of LC using PG**.**

## Discussion

The etiology of LC is complex and so far, no final conclusion was obtained. It is related to smoking, air pollution, dietary factors, decreased immune function and genetic factors (Li and Wang, 2019), while TCM believes that the main cause in the development of LC is the lack of “vital qi”, since the “deficiency of vital qi” is “evil”. Professor Jia Yingjie claims that the “deficiency of vital qi and coexistence of toxin and blood stasis” is the key of the pathogenesis of LC (Yu et al., 2020). PG exerts the effect of smoothing the lung, dispelling the phlegm, and expelling the pus, which is consistent with the pathogenesis of LC. However, the mechanism of PG in the treatment of LC is still unclear due to the multi-ingredient and multi-target characteristics of TCM. Therefore, it is imperative to analyze the ingredients of PG and explore the mechanism used by PG in the treatment of LC.

In this study, 47 non-volatile ingredients were identified from the alcohol extract of PG by the UPLC-Q-TOF-MS/MS technique. According to the ADMET parameters and other basic properties of the ingredients, and combined with the results in the literature, nine main active ingredients including platycodin D and apigenin were selected. The results showed that they play an important role by affecting 285 overlapping genes involved in the treatment of LC. The PPI network showed that the DC values of GAPDH, AKT1 and TP53 were greater than 175, and they were predicted as the most relevant targets. The enrichment analysis of the GO pathway and KEGG pathway in the 20 core targets showed that most of the functional enrichment results of GO were apoptosis, oxidative stress and energy metabolism. Our hypothesis was that oxidative stress response might be the most important biological process managed by PG in the treatment of LC. The results of the KEGG pathway enrichment analysis showed that the top 10 pathways included cancer pathway, TNF signaling pathway, MAPK signaling pathway, P13K-AKT signaling pathway and FoxO signaling pathway. Among them, MAPK signaling pathway, PI3K-AKT signaling pathway, FoxO signaling pathway and HIF-1 signaling pathway were related to cell proliferation and apoptosis, oxidative stress and inflammation. MAPK signaling pathway is one of the most important pathways regulated by PG in the treatment of LC. Related studies showed that the MAPK signaling pathway participates in the regulation of cell cycle, apoptosis and proliferation of NSCLC cells, and inhibits the expression of P-glycoprotein (P-gp), multidrug resistance gene 1 (MDR1), TP53 and other proteins, thus inhibiting the growth of NSCLC cells, blocking cell cycle and inducing apoptosis (Liao et al., 2020; Qiao et al., 2020; Wang et al., 2020). PI3K-AKT signaling pathway is considered as the primary pathway for cancer cell survival, since it promotes tumor cell proliferation and metastasis, and inhibits apoptosis and angiogenesis. If this pathway is abnormal, it directly leads to the abnormal proliferation of cells (Ma et al., 2014; Sui et al., 2018). Therefore, the activation of the MAPK signaling pathway and the inhibition of the PI3K-AKT signaling pathway are crucial in the treatment of LC.

The D-I-T-P-D network showed that the same target could interact with many ingredients. For example, PTGS2 could bind ferulic acid, luteolin, linoleic acid, apigenin and sophorin, while CASP3 can bind methyl linolenic acid, apigenin, ferulic acid and robinin, indicating that multiple active ingredients in PG might act on the same target. In addition, our results showed that apigenin was related to CASP3, AKT1, FOS, JUN, MAPK8, TNF, VEGFA, and CCND1, and linoleic acid was related to PTGS2, TP53, ESR1, MAPK1, MAPK8 and IL6, which was consistent with the results obtained by molecular docking, suggesting that PG could act on multiple targets through the same active component. This result also explained the characteristics of multi-ingredient and multi-target synergism of PG, providing a basis in the mechanism of PG to treat LC. Our hypothesis was that PG was closely related to cell proliferation and apoptosis in the treatment of LC according to the GO pathway and KEGG pathway enrichment analysis. Several core components of PG could interact with PTGS2 and CASP3, potentially promoting cancer cell apoptosis through specific signaling pathways such as MAPK signaling pathway and P13K-AKT signaling pathway in order to cure LC.

The survival analysis results showed that the top five core targets (GAPDG, AKT1, TP53, IL6, and MPKA3) in the PPI network were closely related to the survival of LC patients. The overall survival of LC patients with a high expression of these genes was significantly lower than that of patients with their low expression, suggesting that its overexpression is related to a poor prognosis in LC patients. Thus, they could be used as biomarkers to evaluate LC prognosis.

The results of molecular docking showed that apigenin, luteolin, linoleic acid, kaempferol and methyl linolenate might be the potential active ingredients. Some studies showed that apigenin has a cytotoxic effect on human LC cisplatin-resistant cell line A549/DDP, and indeed it effectively inhibits its growth and reverse its drug resistance (Zhao et al., 2017; Mo et al., 2020). Zhou Liang et al. found that luteolin inhibits the metastasis and proliferation of LC through the down-regulation of the PI3K/AKT signaling pathway and the improvement of the immune function in the body (Zhou et al., 2017). In addition, Li Xiaolin et al. demonstrated that luteolin effectively blocks A549/DDP cell cycle and even promotes apoptosis, and that TP53 protein is involved in this process (Li et al., 2009). In addition, some scientists found that kaempferol weakens the invasion and migration ability of NSCLC A549 cells by inhibiting the expression of estrogen-related receptor *α* (ERR*α*), thus providing a strong support in the study of the anti-cancer mechanism of kaempferol (Zhang et al., 2018). However, Mouradian and other scientists found that linoleic acid increases the activity of PI3K/AKT signaling pathway, promotes the proliferation of LC cells, and leads to tumor formation, with GAB1 as the main target of linoleic acid (Mouradian et al., 2014). Apigenin, luteolin, linoleic acid, kaempferol, and methyl linolenate are widely found in plants, but plants that contain all these five components are rare. Besides PG, Panax notoginseng (Liu, 2019) and peony seed (Zhang, 2016a) also contain these five components. However, so far, no reports are available regarding Panax notoginseng and peony seed anti-LC effect. Therefore, our analysis of the effect and mechanism of PG in the treatment of LC is of great significance.

## Conclusion

PG is one of the most commonly used TCM in clinical practice, since it has a significant therapeutic effect on lung-related diseases and has been used in China for thousands of years. In this study, the chemical constituents of PG were analyzed and identified. A total of nine active PG components and 285 overlapping targets of PG and LC were screened. PPI network, GO and KEGG enrichment analysis showed that the mechanism of PG in the treatment of LC might be related to its involvement in cancer cell apoptosis, inflammation and oxidative stress through the MAPK signaling pathway and P13K-AKT signaling pathway. The network pharmacological method developed in this study provided another strategy for a comprehensive understanding of the mechanism of PG in the treatment of LC.

## Data Availability

All datasets presented in this study are included in the article/Supplementary Material.
